# Mutant resources for functional genomics in *Dictyostelium discoideum* using REMI-seq technology

**DOI:** 10.1186/s12915-021-01108-y

**Published:** 2021-08-24

**Authors:** Nicole Gruenheit, Amy Baldwin, Balint Stewart, Sarah Jaques, Thomas Keller, Katie Parkinson, William Salvidge, Robert Baines, Chris Brimson, Jason B. Wolf, Rex Chisholm, Adrian J. Harwood, Christopher R. L. Thompson

**Affiliations:** 1 Centre for Life’s Origins and Evolution, Department of Genetics, Evolution and Environment, University College London, Darwin Building, Gower Street, London, WC1E 6BT UK; 2 Cardiff School of Biosciences, Cardiff University, Hadyn Ellis Building, Maindy Road, Cardiff, CF24 4HQ UK; 3 Division of Developmental Biology and Medicine, Faculty of Biology, Medicine and Health, The University of Manchester, Michael Smith Building, Oxford Road, Manchester, M13 9PT UK; 4 Milner Centre for Evolution and Department of Biology and Biochemistry, University of Bath, Claverton Down, Bath, BA2 7AY UK; 5 Feinberg School of Medicine, Northwestern University, Chicago, Illinois 60611 USA

**Keywords:** *Dictyostelium discoideum*, Functional genomics, Genome-wide mutant resource, Parallel phenotypic analysis, REMI-seq

## Abstract

**Background:**

Genomes can be sequenced with relative ease, but ascribing gene function remains a major challenge. Genetically tractable model systems are crucial to meet this challenge. One powerful model is the social amoeba *Dictyostelium discoideum*, a eukaryotic microbe widely used to study diverse questions in the cell, developmental and evolutionary biology.

**Results:**

We describe REMI-seq, an adaptation of Tn-seq, which allows high throughput, *en masse*, and quantitative identification of the genomic site of insertion of a drug resistance marker after restriction enzyme-mediated integration. We use REMI-seq to develop tools which greatly enhance the efficiency with which the sequence, transcriptome or proteome variation can be linked to phenotype in *D. discoideum*. These comprise (1) a near genome-wide resource of individual mutants and (2) a defined pool of ‘barcoded’ mutants to allow large-scale parallel phenotypic analyses. These resources are freely available and easily accessible through the REMI-seq website that also provides comprehensive guidance and pipelines for data analysis. We demonstrate that integrating these resources allows novel regulators of cell migration, phagocytosis and macropinocytosis to be rapidly identified.

**Conclusions:**

We present methods and resources, generated using REMI-seq, for high throughput gene function analysis in a key model system.

**Supplementary Information:**

The online version contains supplementary material available at 10.1186/s12915-021-01108-y.

## Background

The past two decades have seen an exponential increase in genome sequence availability. However, our ability to comprehensively ascribe function to gene sequences is still limited. One solution is to identify and characterise the effects of loss of function mutations in all genes across the whole genome. When coupled with methods that allow high throughput characterisation of these mutants, this has the potential to provide system-level genotype-phenotype information. Consequently, efficient tools for random or systematic genome-wide mutagenesis are crucial and major efforts have been made to develop these tools in model organisms. However, genome-wide functional analyses are only possible in a handful of higher eukaryotes. This is because large diploid genomes provide an obstacle to generating extensive mutant collections. Furthermore, large numbers of individual mutant lines must be generated and then bred to homozygosity, or alternatively strains that express inhibitors or activators of gene function must be generated. Finally, phenotypic analyses are experimentally challenging as individual strains often must be grown up and examined. Whilst libraries of cell culture mutants provide a potential solution, this in turn limits the spectrum of phenotypes that can be studied.

Eukaryotic microbial model systems provide a solution to many of these problems. First, some have small haploid genomes, which allow libraries of thousands of ‘barcoded’ single-gene loss or gain-of-function mutants to be generated by homologous recombination [1] or large-scale random insertional mutagenesis [2–5]. Second, their fast generation times and relatively simple phenotypes allow genome-wide forward genetics approaches in which these libraries of mutants can be screened under different selection regimes to enrich for advantageous mutations or deplete disadvantageous mutations. In recent years, massively parallel sequencing has allowed the frequency of every mutant allele to be quantified in a library before and after a selection [6]. Such parallel phenotypic approaches have proven a powerful tool to study diverse biological processes, including the definition of genes required for growth, survival under different conditions, virulence and antibiotic resistance, as well as for genetic interaction discovery [3, 7–11].

One drawback of simple microbial systems is that they often cannot be used to study multicellular traits characteristic of higher eukaryotes, such as cell differentiation, cell-cell signalling and cell migration. However, the eukaryotic microbial model system, *D. discoideum* provides a potential solution. It is already widely used for studying diverse processes in cell, developmental and evolutionary biology because it exhibits a unique lifecycle, with both unicellular and multicellular stages [12]. During unicellular growth, it is motile, actively seeking and engulfing bacteria via actin-mediated locomotion and phagocytosis. It divides by binary fission, again using an actin mediated process, and can grow in a liquid medium by upregulation of macropinocytosis. Upon starvation, it initiates a programme of multicellular development, where approximately 100,000 cells aggregate by chemotaxis to cAMP. As it enters its multicellular state, cell-cell signalling controls the temporal and spatial patterning of a small number of different cell types. Ultimately, these cells terminally differentiate to form a fruiting body, composed of dead stalk cells that aid the dispersal of viable spores. As a consequence, the *D. discoideum* life cycle provides a powerful model for studying fundamental cell processes, such as cell motility, chemotaxis [13], macropinocytosis and phagocytosis [14, 15]; as a single-celled host for a large number of intracellular bacterial pathogens [16], as well as aspects of mutualism and pathogenesis during multicellular stages [17, 18]; as a model for cell-cell signalling, differentiation and morphogenesis during multicellular development [19]; and longer term evolutionary changes due to social conflict [20–25]. Unsurprisingly, sequencing of the compact haploid (34Mbp) genome of the widely used laboratory strain AX4 reveals widespread gene conservation with higher eukaryotes [26]. Finally, random mutagenesis by restriction enzyme-mediated integration (REMI) insertion [27, 28] has been developed to allow forward genetic identification of novel components of different biological processes [29–36].

Many studies illustrate the potential of *D. discoideum* as a model for understanding conserved biological processes. However, its true power for functional genomics has yet to be harnessed. Genomic, proteomic and transcriptomic studies can be performed with relative ease, but testing the functional importance of identified genes is challenging due to a lack of a genome-wide collection of mutants. Individual mutants can be made by gene targeting via homologous recombination [37–39] or CRISPR/Cas9 technology [40]. However, such gene-by-gene approaches are time-consuming and resource-intensive. Large-scale generation of REMI mutants has been possible for many years, but has been limited by the need to identify individual insertion sites after genetic screens [35, 41]. To address these problems, we describe an adaptation of Tn-seq [3], REMI-seq, that allows high throughput parallel identification of REMI insertion points and its use to generate near genome-wide resources which have been made freely available for the *D. discoideum* research community.

## Results

### REMI can be used to generate mutations in most *D. discoideum* genes

**Fig. 1:**
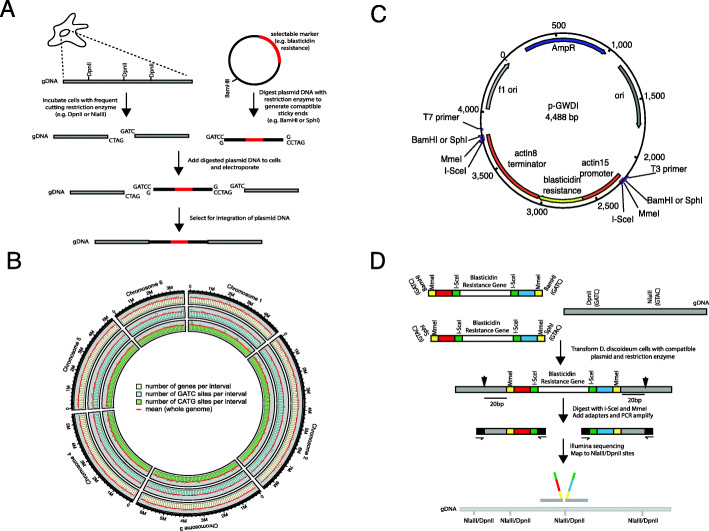
REMI-seq can be used to generate a comprehensive mutant collection in *D. discoideum*. **A** Schematic illustration of REMI mutageneisis. Insertion will take place if DpnII is used as the mutagenic enzyme together with BamHI digested linear plasmid. This will also work with NlaIII if the BamHI site is replaced with a SphI site. **B** In silico analysis suggests random genome-wide accessibility to REMI mutagenesis using a combination of restriction enzymes. Predicted DpnII and NlaIII sites are densely and randomly distributed across the genome. The density of each site was mapped onto 10kb fragments of the genome. Few regions are under- or overrepresented and thus represent putative cold or hotspots. **C** Schematic of the REMI-seq vector. pGWDI is a derivative of pLPBLP which encodes the blasticidin *S deaminase* selectable marker under the control of the actin15 promoter and actin8 terminator sequences. The sequence was modified (purple box) to contain sequences for REMI insertion. The pGWDI-C and pGWDI-G plasmids are identical with the exception that they have SphI and BamHI sites for integration. In addition, four different versions of each vector were generated with unique left and right 6bp sequence indices to allow increased multiplexing for insertion point analyses. **D** Schematic of insertion identification by REMI-seq. The pGWDI fragment is introduced in the *D. discoideum* genome by electroporation in the presence of DpnII or NlaIII. The termini of the insertion cassette contain recognition sites for MmeI and I-SceI which allows 20 bp fragments of gDNA at the insertion site to be generated. Red and blue boxes indicate vector-specific barcodes that allow multiplexing. Following the addition of adapter sequences, PCR amplification and Illumina sequencing, mapped reads can be aligned to the reference genome to identify insertion sites. Index sequences can be used to identify orientation/quantify the number of reads that map to each site provides quantitative information about the mutant abundance

Genome-wide collections of mutants are useful for researchers seeking either to ascribe gene function by reverse genetics experiments on individual clones or unbiased forward genetic screens on pools of mutants. REMI [29–36] potentially provides a method to generate large collections of *D. discoideum* mutants. Cells are briefly incubated with a restriction enzyme that cuts genomic DNA to leave a four-base pair overhang. A plasmid that has been cut with an enzyme that generates compatible four base pairs overhand is then added, and cells are electroporated to induce plasmid uptake. Integration of the plasmid within genomic DNA can be selected because it carries a selectable drug resistance marker (Fig. 1A). Different combinations of mutagenic enzyme and compatible restriction sites within the plasmid allow different genomic sites to be targeted. To test whether REMI could be used to generate a genome-wide collection of REMI mutants, we performed in silico analyses to determine how many genes are accessible to REMI with different mutagenic restriction enzymes (DpnII (GATC) and NlaIII (CATG)). These were chosen because the *D. discoideum* genome is extremely AT biased, with coding regions 40% GC and non-coding sequences less than 10% GC. Consequently, these sites are overrepresented in intragenic sequences [27], with 58,852 out of 69,422 (85%) of DpnII sites and 41,845 out of 49,365 (85%) of NlaIII sites found within coding regions of genes (Additional file 1). Moreover, >92% of the annotated genes can be targeted using a combination of the two insertion vectors. From a total of 13,412 putative genes, 12,391 genes contain one or more DpnII or NlaIII sites (or both) (Additional file 1). Furthermore, of the remaining 1021 genes containing neither site, 496 contain restriction sites in their respective promoter regions (< 500 bp upstream of the start codon). Of the remaining 525 genes, 116 are tRNAs, 28 are small non-coding RNAs and another 35 are pseudogenes or transposable elements. Finally, these sites are uniformly distributed across the genome (Fig. 1B), with mean numbers of 202 GATC and 143 CATG sites per 100,000 bp interval. Only four and three intervals show a significantly higher number of DpnII or NlaIII sites respectively (3 standard deviations higher than the mean) and thus represent putative hotspots.

The in silico findings reveal DpnII and NlaIII should allow a genome-wide collection of REMI mutants to be generated. We thus designed a series of plasmids (pGWDI) (Fig. 1C, Additional file 2) that could be used to target NlaIII (CATG site; pGWDI-C) or DpnII (GATC site; pGWDI-G) sites. To enable insertion sites to be identified in an efficient and inexpensive fashion, we adapted Tn-seq methods that have allowed high throughput identification of insertion sites in organisms amenable to transposon mutagenesis [3, 6]. For this, pGWDI plasmids were engineered to contain recognition sequences for the type IIS restriction enzyme MmeI, which cuts 19–20 bp downstream of the recognition site. Furthermore, cut sites for the meganuclease I-SceI (which does not appear in the *D. discoideum* genome) were positioned 3’ to each MmeI site (Fig. 1D). Consequently, when genomic DNA is digested with MmeI, it will cut within the genomic DNA flanking the site of insertion (Fig. 1C), and subsequent digestion of genomic DNA with I-SceI will result in the generation of two 47 bp fragments. These fragments can be enriched by PCR, purified and sequenced by Illumina sequencing (Fig. 1D) [3]. Each read contains a 28 bp sequence comprised of the I-SceI and the MmeI restriction sites, vector identifier index sequence, the remnants of the DpnII or NlaIII site, and importantly an additional 19-20 bp of genomic sequence derived from the site of insertion. Even though the tags are fairly small, in silico analysis of the *D. discoideum* genome revealed that most genes (11,244 out of 13,421) have at least one uniquely identifiable tag (Additional file 1). This method, termed REMI-seq, thus has the potential to allow the high throughput generation of a near genome wide insertion mutant resource in *D. discoideum*.

### GWDI-bank: isolation and identification of large numbers of *D. discoideum* REMI mutants

**Fig. 2:**
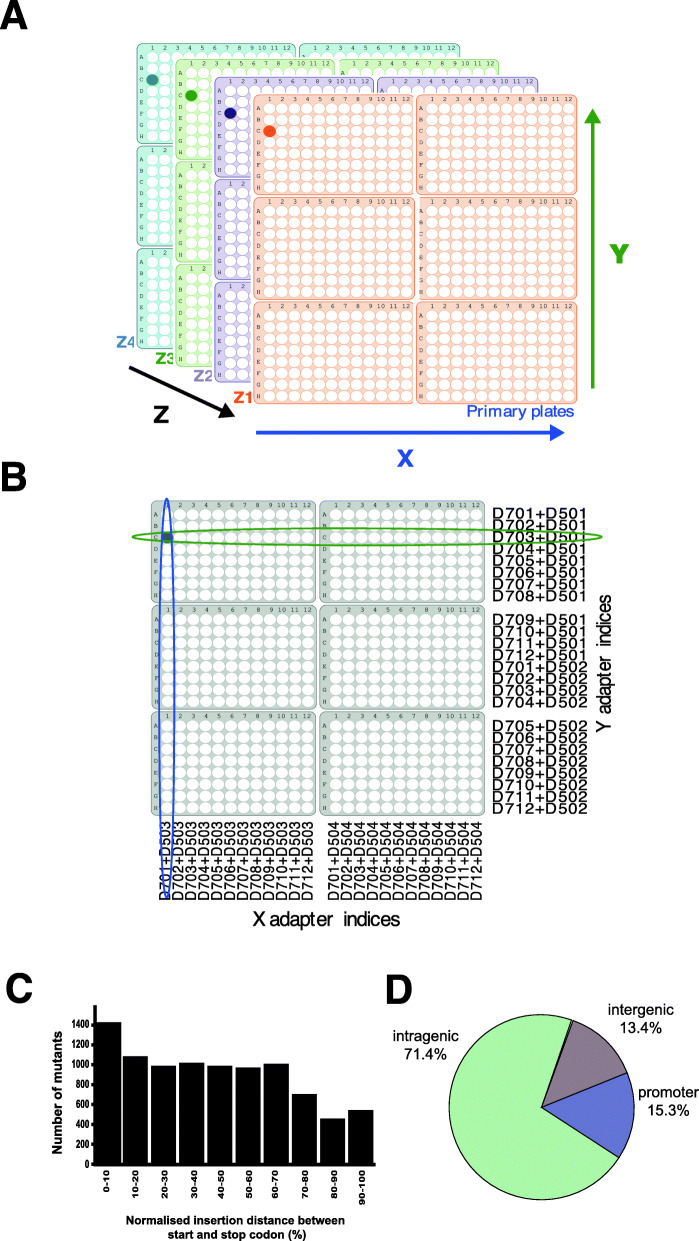
Gridding and multiplexing of clonal mutants for sequencing. **A** Master grid contains 2304 wells. A grid containing six plates of mutants with the same barcode was generated. This was repeated for mutants with each GWDI barcode to make a stack of four grids. **B** Multiplexing of mutants for sequencing. Cells in each row (X), column (Y) and stack (Z) were pooled and gDNA prepared, to make a grid that is compressed into the X and Y coordinates. Unique combinations of X and Y barcoded sequencing adapters were ligated to each gDNA preparation before Illumina sequencing. The intersection of common mutants provides the well location for each mutant. The four mutants present in the compressed grid can be separated into stacking grids by their unique Z-barcode. **C** Intragenic insertions are biased towards the 5’ end of genes. The distance of each intragenic insertion from the start and stop codon was calculated and normalised to gene length as a percentage. 0% is the position of the start codon and 100% is the position of the stop codon. **D** The collection of individual *D. discoideum* REMI mutants. The REMI-seq mutant resource consists of 21,529 individual mutants. The majority of these mutations are in coding sequence or within putative promoter sequences and thus likely to result in gene disruptions. The REMI-seq resource consists of mutations in 5705 genes, resulting in a large increase in the number of mutants available. The catalogue of mutants can be found on the REMI-seq website (remi-seq.org) and individual mutants can be ordered from the *Dictyostelium* stock centre

The number of mutants that are currently available in *D. discoideum* represents only 5–10% of the total genome. Furthermore, these mutants were made in a variety of different genetic backgrounds, making comparisons difficult. We therefore developed a pipeline (Fig. 2) to generate a genome-wide collection of gridded mutants in the same isogenic background. For this, we first developed REMI protocols that worked efficiently for two different restriction enzymes. PCR was used to generate integration DNA fragments containing a blasticidin resistance cassette from pGWDI plasmids, which were then introduced into the *D. discoideum* cells together with the appropriate accompanying restriction enzyme. The widely used laboratory strain AX4 was chosen as this was used to generate the reference genome, which ensures that experimentally observed insertion sites can be accurately matched to in silico predicted sites. It also reproducibly gave high rates of transformation across multiple independent transformations, which allowed us to generate >35,000 transformants. Mutant cells were plated at limiting dilution in 96-well dishes to favour clonal growth. After growth for 1–2 days, wells containing a single mutant were transferred into 368 master plates. Each plate was replicated to allow cells to be used for DNA extraction (to identify insertion points) and to provide stocks for mutant strain storage.

Mutant identification was based on a conceptual 24×24 grid, the equivalent of a 2 × 3 array of 96-well plates (Fig. 2B). Mutants from each row and each column were pooled and their DNA extracted (Fig. 2B). In this way, each mutant is represented twice, once in a row (X) and once in a column (Y), and can be identified by coordinating its XY position within the 24 × 24 notional grid. To do this, a series of 16 library adapters (D501-D504 and D701-D712) required for Illumina sequencing were made that introduce a unique 8bp barcode to each fragment terminus (Additional file 3). A further grid dimension was added with a *Z* coordinate, corresponding to a notional stack. Each layer of the stack was identified by using a different insertion fragment, again each marked by a unique barcode (Additional file 3). After DNA sequencing, the 3D-grid position of each mutant can be inferred computationally by its unique XYZ coordinate (Fig. 2B, C). Consequently, each sequencing run had the capacity to generate information from 4608 wells or mutants.

DNA sequences, both upstream and downstream, of all integration sites, were identified by first filtering the read files for the presence of short sequences derived from the insertion fragment and then aligned to the *D. discoideum* AX4 reference genome (Additional file 1). We were able to assign 21,529 independent mutants, corresponding to 12,247 different genomic insertion sites (Additional file 4). We termed this resource the GWDI bank. From these, we were able to catalog 12,247 different insertion points, of which 8739 (71%) were found to lie within coding regions of genes. The vast majority of these are likely to result in gene disruption, as we observed a systematic bias towards insertions towards the 5’ end of genes, with 3’ insertions, which may have a lesser effect, less represented (Fig. 2C). Little difference was seen in the spectrum of genes targeted by each enzyme, with >93% of genes disrupted by NlaIII also disrupted by DpnII (albeit necessarily giving a different spectrum of alleles). In addition, targeting with these enzymes revealed another 1872 insertions within putative promoter regions of genes (defined as <500 bp upstream of the start codon) and thus also likely to disrupt gene function (Fig. 2D). In total, our REMI-seq approach has resulted in the production of a collection of mutants in 5704 different genes (i.e. 43% of the genome).

During this process, each additional sequencing run generated diminishing returns in terms of the number of new gene disruptions identified. This could reflect the fact that some genomic regions or genes are hot or cold spots for insertion. Alternatively, because sites in essential genes will likely largely be refractory to REMI mutagenesis, this could suggest our mutagenesis may be close to saturation. To address this, we first performed a genome-scale analysis of insertion bias. In silico genome analyses, using a window size of 40,000 bp, which divides the 35 Mbp genome into 861 non-overlapping windows, predicts few hot or coldspots. Consistent with this idea, when the number of possible sites per window was compared to the number of observed insertion points (see the “Methods” section), only 15 out of 861 windows contained fewer (coldspots) and 28 contained more insertion points (hotspots) than expected under a uniform distribution using an adjusted *p* value cutoff of 0.05. Regions associated with hotspots thus only account for 8% of the observed insertions in the pool. This suggests that the overall frequency with which different sites are hit is close to random at a genome-scale. We next analysed the probability of insertions at a single-gene level. For this, genes were grouped according to the number of possible insertion sites. This was used to model a Poisson distribution for the number of times genes would be expected to hit if it is random. In all cases, the number of genes with no insertions was higher than expected from a Poisson process (Additional file 5). This is consistent with the idea that selection for growth alters the probability that some genes are hit. In addition, this effect is likely exacerbated by the fact that a small number of genes (~ 350) are insertion hotspots. Together, these data suggest that the REMI-seq library is likely to be as close to saturation as may be biologically and technically feasible using this technique.

### Use of GWDI-bank in functional genomics pipelines

A number of standard ‘omics’ approaches are available for *D. discoideum* research, such as transcriptomics and proteomics. The GWDI bank has the capacity to integrate across these modalities by connecting genotype to phenotype. To explore this potential, we undertook a pilot experiment based on a proteomics study of actin cytoskeletal-associated proteins [42]. This study used SILAC to identify over 470 proteins that associate with the actin cytoskeleton during chemotaxis. The challenge is to identify those proteins that are regulatory and important for actin cytoskeletal dynamics, and this requires the generation of a mutant in each gene. We, therefore, selected 12 of the proteins identified in this analysis represented by 2 or more independent mutants in the GWDI bank, of which the function of five of the proteins was unknown (Additional file 6).

*D. discoideum* cell motility is driven by F-actin polymerisation, and changes in its regulation are likely to translate into altered cell movement. Almost all *D. discoideum* cell motility assays are based on starved mid-aggregation stage cells undergoing chemotaxis to cAMP or chemotaxis of bacterially grown cells to folate. Developed cells show a significantly higher speed and directionality of movement compared to those in growth phase [43, 44]. This difference may arise due to the influence of different molecular pathways controlling chemotaxis [13]. However, screening for chemotaxis defects in large numbers of mutants is difficult. We therefore devised a simple assay for the motility of growing cells under normal growth conditions. In our assay, cells were plated into a 96-well microtitre plate at a low enough density to allow cell movement to be recorded by video microscopy. Once plated and adhered to the plate surface, individual cell positions were recorded every 2 min for a minimum of 44 min using high content microscopy. The distance travelled between time points was measured, and when summed gives the total distance travelled. This assay allows each mutant to be assayed under identical assay conditions, and its ease of setup offers a future basis for high throughput screening (HTS).

**Fig. 3:**
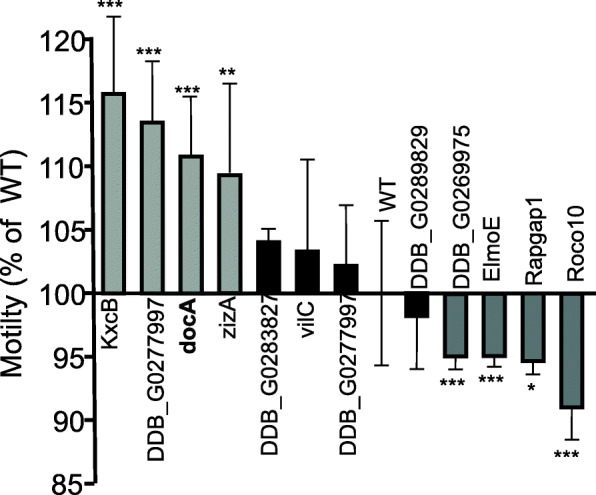
Screening mutant strains for motility changes in growth phase culture. Cells from two independent strains for each mutant were plated in wells of a 96-well plate in parallel cultures. 100–1000 cells were imaged for 44 min. The histogram shows the mean of the total path length for the two strains of each mutant, expressed as a percentage of the wild type control (WT), error bars show standard deviation. *p<0.05, **p<0.005 and ***p<0.001

The motility of the 12 mutants identified through proteomic analysis and selected from the GWDI bank was assayed (Fig. 3). Although individual cells in culture show substantial variation in distance travelled, significant differences were found for some mutants when compared to the wild cells. For example, the DOCK protein-family gene *docA* has been examined in detail previously [42] and found to affect chemotaxis in starved cells. Similarly, we found that it affects motility in slow-moving growth phase cells. We also found ziziminA (zizA), another DOCK family protein shown to localise to the microtubule organising centre (MTOC), but with no reported phenotype [45] has elevated motility in this assay. Finally, we tested another DOCK protein *docB*, which is represented as a single-mutant cell strain in GWDI bank and also found it to exhibit increased motility. In contrast, we found that *elmoE* showed reduced motility, as did *roco10* and *rapgap1*. The ELMO family of proteins forms a complex with DOCK proteins to function as Rac-GEF. In *D. discoideum,* loss of *elmoE* reduces cell speed and shows reduced activation in response to cAMP stimulation [46]. Loss of *rapgap1* is also known to affect cell motility deficits during chemotaxis, and here, we show that this may be the case for non-chemotactic growth phase cells [47]. In addition, our assay showed that previously untested gene mutations of roco10, DDB_G0289829 and DDB_G0277675 all reduced cell motility, while DDB_G0277997 encoding a protein of unknown function elevated motility. This pilot experiment thus demonstrates that a simple phenotype can be employed to screen GWDI-bank and connect the output of proteomic studies to gene function.

### GWDI-pool: a pooled library of mutants for parallel phenotypic forward genetic screens

**Fig. 4:**
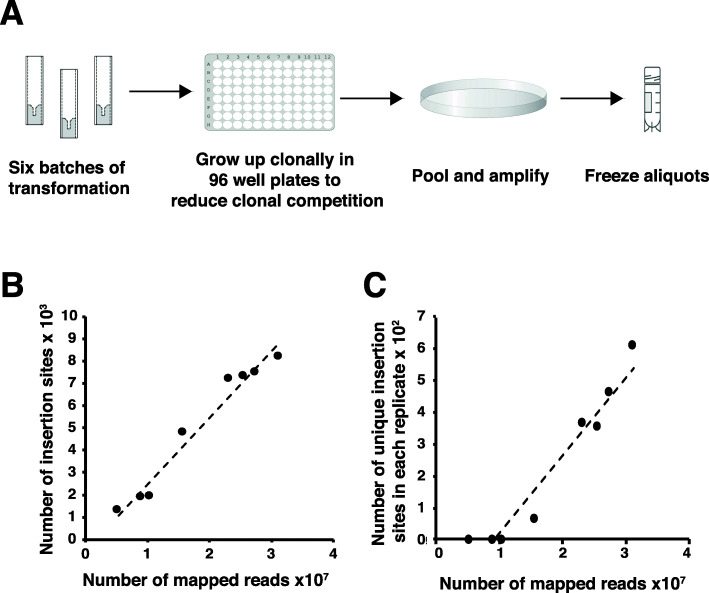
Generation of a pooled REMI library for parallel phenotypic analyses. **A** Schematic of pooled library generation. REMI transformations were plated out at limiting dilution to ensure clonal growth and avoid competition between clones. After clonal growth, mutants were pooled to generate frozen stocks that are available from the *Dictyostelium* stock centre. **B**, **C** Independent replicas of the pooled library are highly similar. Eight independent replicas of the pooled library were grown up and sequenced. Although the total number of insertion sites (**B**) and insertion sites that are unique to each library (**C**) is different, this is can be wholly accounted for by sequencing depth

‘Parallel phenotypic’ approaches provide a powerful method to determine the relationship between genotype and phenotype [6, 48], by establishing mutants that confer a selective advantage or disadvantage within the population under different conditions. The power of such an approach depends on the ability to generate sufficient numbers of mutants for comprehensive screening, as well as the ability to quantify the relative abundance of these variants over the course of a selection. In order to allow other researchers to perform parallel phenotypic analyses, we wanted to generate a ‘standard’ high quality and complex pool of REMI mutants (GWDI-pool) that would be made freely available. A REMI pool was generated in the laboratory strain AX4 from six independent transformations that were first grown-up clonally to minimise competition, before amplification and storage (Fig. 4A). Because this GWDI-pool is the start point against which any selection regime will be compared, it was grown up and sequenced independently eight times to ensure that it exhibits reproducible complexity after storage. This revealed that the complexity does not differ between replicates. The total number of insertion points identified and the number of insertion points that are unique to each replicate are strongly correlated to the depth of sequencing (Fig. 4B, C, Additional file 7). Consequently, this means that differences in complexity can be accounted for by sequencing depth and that this represents the major limiting factor. The REMI-seq method is thus quantitatively reproducible as read numbers across different biological replicates are strongly correlated (Fig. 4B, C, Additional file 7) and can be used to monitor the relative frequency of large numbers of mutants within a mutant pool.

The reproducibility of data from these replicates allowed the sequencing data to be pooled to generate an accurate picture of library composition from an extremely high depth of sequencing (a total of almost 150M mapped reads) (Additional file 7). These data reveal the library contains insertions at 10,499 different DpnII or NlaII sites. Of these, we found that these comprised 7,207 coding sequence insertions, resulting in the disruption of 4,482 different genes. A further 1,700 intergenic mutations lie within promoter sequences (within 500 bp of the start codon). Together, this equates to the mutation of 5438 genes when promoter insertions were included (41% of all genes in the genome) (Additional file 7). These are composed of 8758 individual mutations, and thus for most genes, there are multiple alleles. In fact, the number of independent mutants is far higher than this when the six different vectors used to make the library are separated, or when the orientation of insertions is taken into account (although this decreases the read coverage for each mutant).

### Identification of mutants that exhibit fast or slow growth

In all parallel phenotypic experiments, a library of mutants is grown up and then placed under a selective pressure of interest. The remaining mutants are typically regrown and rounds of selection and growth are repeated. Consequently, some growth of the *D. discoideum* REMI pooled library, either on bacteria or liquid medium, will always be required for researchers to perform parallel phenotypic experiments. Consequently, in order to identify mutants that are enriched or depleted due to the selection regime, researchers must distinguish these from mutants that simply grow faster or slower. To facilitate this process, we have performed extensive selection experiments to identify and catalog mutants that exhibit differences in growth rate.

**Fig. 5:**
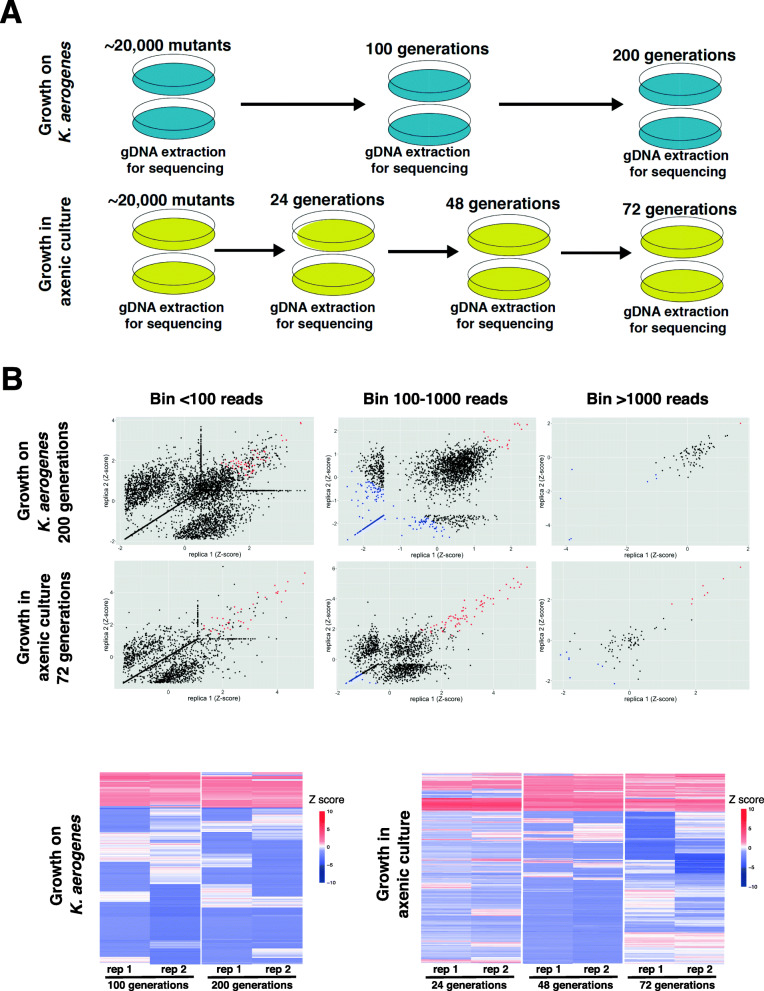
REMI-seq parallel phenotyping to identify mutants that affect axenic or bacterial growth. **A** Schematic of selections to identify mutants with growth advantages and disadvantages. A pool of ~20,000 mutants was plated in duplicate in HL5 medium for axenic growth or in association with *K. aerogenes* (Ka) bacteria. Cells were grown for 24 (axenic) or 100 generations (Ka) before a sample was harvested for sequencing. The remaining cells were diluted and grown-up for a further 24 (axenic) or 100 (Ka) generations before a second sample was taken for sequencing. Axenic cells were diluted and grown-up for a further 24 generations before sequencing. **B** Identification of mutants with growth advantages and disadvantages. The abundance (read count) at each round of each mutant was compared to the start pool. Mutants were first divided into bins based on their read count in the start pool in order to identify mutants that deviated in abundance (*Z* score) significantly from other mutants with similar read counts. Mutants with a mean *Z* score of >1.5 and with a minimum of 100 reads in each replicate at the end of the selection were considered to have increased in abundance (red points). Mutants with a mean *Z* score of < -1.0 (blue points) were considered to have decreased. In bin <100, the variation due to technical dropouts resulted in a high false discovery rate, and mutants that decreased were not considered. Replicas are highly correlated. Results are shown at the end point of the selection (after 200 generations of Ka growth and 72 generations of axenic growth). Data from other rounds showed similar patterns (see Additional files 8 and 9). **C** Mutant behaviour is consistent across rounds of selection. Mutants were identified with a significant advantage or disadvantage at each stage of the selection. The level of enrichment or depletion (deviation from expected or *Z* score) was plotted for each mutant at all rounds

The GWDI-pool was selected for growth either on the standard bacterial laboratory food source *K. aerogenes* or under axenic conditions in liquid HL-5 media. Growth on bacteria requires phagocytosis of particles [14] whereas growth in liquid medium requires macropinocytosis of fluid [49, 50], and therefore a different spectrum of genes are likely to underpin each mechanism. The population was serially passaged in duplicate through growth either in HL-5 axenic culture for up to 72 generations or in association with *K*. *aerogenes* over 200 generations (Fig. 5A). The samples were sequenced after 24, 48, and 72 generations of axenic growth or 100 and 200 generations of growth on *K*. *aerogenes* (Additional file 8). As expected, the read counts of each biological replicate were highly correlated (*p* ≤10^-15^, Additional file 8), and the amount of technical variation was dependent on the initial read counts (Additional files 7 and 8). To determine if mutants became under- or overrepresented, we compared the relative read count of each mutant in the starting library to the relative read count after growth selection. Mutants were first separated into the three bins (1–100, 100–1000, >1000 reads) according to their read count in the starting library. This allowed us to determine the average change in behaviour of each mutant within each bin to identify those mutants whose abundance deviated significantly (*Z* score) from this behaviour (Fig. 5B and Additional files 8, 9 and 10). This takes into account that mutants with increased fitness will increase in frequency and alter the relative abundance of neutral mutants (see Additional files 8, 9 and 10). This is especially evident in the stronger axenic growth selection, where the number of reads for neutral mutants decreases substantially with many disappearing altogether (Additional file 8). In addition, mutants with initial read counts of <100 that decreased in frequency were discarded from the analysis. This is because the technical dropout rate in this group is very high (Additional files 8, 9 and 10). Together, these steps allowed the identification of mutations that significantly affect fitness during the selection (Additional file 11).

Our analyses revealed that a significant proportion of mutants that are enriched or depleted in one round of the selection were often also enriched or depleted in future rounds (Fig. 5C). As expected, few mutants switched from being significantly enriched to depleted, or vice versa. Some mutants, however, were only enriched or depleted in early or late rounds of the selection. This is likely due to the complex dynamics of selection. For example, weakly advantageous mutations may be lost in later rounds as stronger mutants take over the population. Alternatively, the same mutants could take several rounds to become significantly enriched. Similarly, it is sometimes impossible to determine if mutants are significantly depleted in later rounds if all mutants in the bin as a whole have essentially been removed (i.e., mutant frequencies cannot decrease below zero). These data thus illustrate the importance of observing multiple rounds over the course of a selection to capture the full spectrum of phenotypes within the population.

**Fig. 6:**
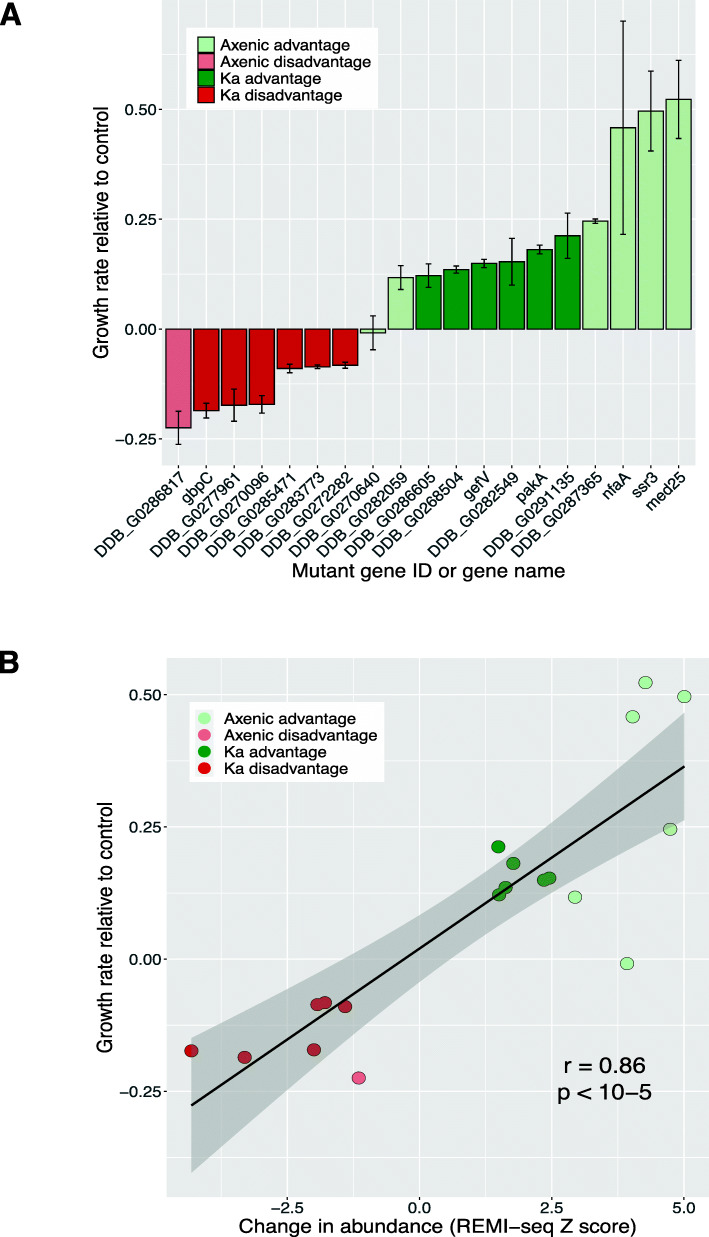
Validation of mutants identified by REMI-seq. **A** Mutants identified by REMI-seq exhibit growth advantages or disadvantages. Independent isolates of 19 mutants identified by REMI-seq were tested for growth effects in competition with the parental AX4 strain. 18 mutants showed significant differences, and all mutants identified by REMI-seq as disadvantaged grew more slowly than wild type. **B** REMI-seq can quantitatively predict fitness effects in growth competition. There is a strong correlation between the experimentally measured growth rate of each mutant and the relative change in abundance of each mutant inferred by REMI-seq at the final round of selection. Similar results can be seen when the data from every round is compared to the growth rate (see Additional file 10)

To confirm that the gene mutations identified from parallel phenotyping do indeed confer positive or negative fitness under the different growth conditions, we tested independently generated mutations at the same locus isolated from the gridded GWDI-bank. The fitness of each mutant was validated in 1:1 competition with their parental strain (Fig. 6A). Consistent with expectations, fitness in 1:1 competition was highly correlated with fitness measured in the equivalent population screen (Fig. 6B and Additional file 12).

### Functional genomics of axenic and bacterial growth

**Fig. 7:**
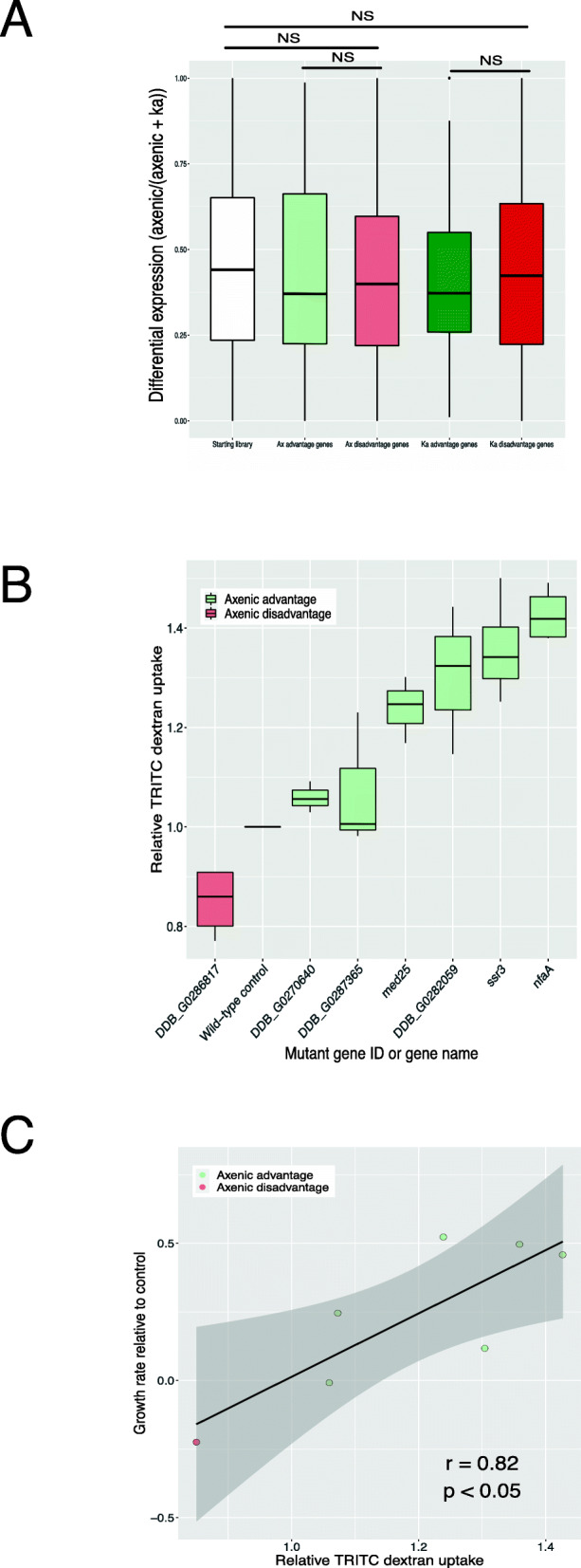
REMI-seq identifies novel regulators of axenic and bacterial growth. **A** Differential expression does not correlate with gene requirement. Gene expression was compared in cells grown in an axenic medium or in association with *K. aerogenes*. Differential expression was calculated as an expression index where a value of 1 represents expression exclusively in axenic growth and a value of 0 represents expression solely in bacterial growth. The expression index of genes in which mutations result in increased or decreased growth rate in axenic or bacterial growth was compared to all other mutations in the library that could be ascribed to the likely functional disruption of a single gene. No significant differences were found. **B** The rate of macropinocytosis is affected in mutants with altered growth in axenic medium. The rate of TRITC-dextran uptake was compared in a selection of seven mutants identified in the axenic growth selection to parental AX4 wild-type cells. The rate of uptake was significantly different from wild type in six mutants. **C** The rate of macropinocytosis correlates with growth rate in axenic medium. The growth rate in completion with wild type cells (see Fig. 6A) was compared to the rate of TRITC- dextran uptake

The data from growth selection in liquid media or in association with bacteria not only provide a useful resource, but also shed light on the biological processes underlying growth on bacteria or in liquid medium. This is because parallel phenotyping using REMI-seq provides a method to directly address gene function in the absence of other information. In contrast, many other functional genomic approaches infer gene function from correlated changes, such as transcript abundance. We, therefore, compared genes that affect growth on bacteria or liquid medium identified by parallel phenotypic selection to those which would be inferred to play a role due to differential expression (upregulation or downregulation) when cells grown on bacteria or liquid medium are compared. For this, we compared RNA-seq data from cells which had been grown for 2 days either on bacteria or in an axenic medium [51, 52]. Differential gene expression analyses identified genes that were upregulated or downregulated when bacterial or axenic grown cells were compared (Additional file 13). However, we found no correlation between these differentially expressed genes and the gene set identified by REMI-seq (with insertions within the coding sequences or promoter regions of genes) (Fig. 7A). This provides further support for the idea that the relationship between gene function and gene expression may be complex and non-linear. For example, studies in *Saccharomyces cerevisiae* [53] also revealed a surprising lack of correlation between differential transcriptional regulation and a requirement for fitness under those conditions, where fewer than 7% of upregulated genes were also required for optimal growth [1].

One reason for this lack of correlation could be that differentially expressed genes (DEGs) are actually neutral when mutated. Alternatively, if mutations in these genes result in a strong growth defect under either condition, they could be lost from the REMI-seq starting library before the start of the selection. This is because some growth in HL5 or Ka is actually required for the generation and sequencing of the REMI-seq starting library. Consistent with this latter idea, GO terms associated with the identified DEGs were actually underrepresented in the REMI-seq starting library (Additional file 14), including genes required for the core machineries of import into the cell, vesicle-mediated transport and ribonucleoprotein complex assembly. In addition, any remaining mutants associated with these GO terms tended to have lower than average read counts in the starting library (Additional file 7). These data suggest that for a subset of genes, there is good agreement between gene expression and gene function.

We next further analysed genes that were identified because they displayed a phenotype in the REMI-seq screen, but showed little correlation to the degree of differential expression. These genes are interesting because this would have precluded their identification in transcriptomic studies. Genes that affect growth on bacteria were strongly enriched for processes associated with cellular responses to stress, especially the response to oxidative stress (Additional file 14). These included redox homeostasis and superoxide removal (including thioredoxins, glutaredoxin, and protein disulphide isomerase encoding genes), DNA repair, pteridine production and copper ion transport. The role of ROS production in bacterial growth and killing has been unclear [54]. However, these data suggest the ability of *D. discoideum* cells to withstand oxidative stress is crucial for normal growth [55]. It is also interesting to note the enrichment of terms associated with lipid composition, including genes associated with sphingolipid metabolism. Indeed, it has recently been suggested that lipid composition may play a key role in host-pathogen interactions in macrophages [56], but so far has not been noted in *D. discoideum*. These studies thus highlight the importance of processes such as prey recognition, killing and toxin tolerance in addition to uptake for bacterial growth.

Axenic growth requires cells to take up large volumes of fluid from the surrounding environment by macropinocytosis. Indeed, GO terms associated with fluid uptake are enriched in genes identified through differential gene expression analysis and are underrepresented in the REMI-seq starting library (Additional file 14). However, we also found that selection for growth in the axenic medium resulted in the identification of mutants that were enriched for GO terms associated with signalling, especially proteins that affect phosphorylation such as protein kinases and histidine kinase response regulators (Additional file 11). Furthermore, we found enrichment of mutations in genes encoding putative regulators of small GTPase activity, including *nfaA* (which also encodes a Ras-GAP) and DDB_G0286817 (which encodes a putative regulator of GAP activity). These findings are consistent with the idea that the rate of macropinocytosis can be regulated by environmental cues, such as nutrients or amino acid availability [57] or by changes in Ras activity [50]. We, therefore, tested whether mutants that show increased or decreased rates in growth in HL5 medium, also affect the rate of macropinocytosis. The rate of TRITC dextran uptake was significantly different to wild type in six out of seven mutants tested (Fig. 7B). Furthermore, this correlated strongly with the growth rate of each mutant in HL5 medium (Fig. 7C).

## Discussion

The development of REMI-seq technology has allowed us to generate a gridded library and a complex pool of *D. discoideum* insertion mutants. The laboratory strain AX4 was chosen because it was used to generate the reference genome and thus ensures experimentally observed insertions sites can be accurately matched to in silico predicted sites. This allowed us to identify 21,529 individual different gridded mutants in the GWDI bank. This encompasses mutants in nearly 6000 *D. discoideum* protein-coding genes and thus significantly increases the availability of mutants. One drawback, however, is that the AX4 strain is known to contain a duplication on chromosome 2. This means that approximately 300 genes are likely refractive to mutation or will result in hypomorphic alleles. The development of CRISPR technology in *D. discoideum*, however, provides an opportunity to relatively inexpensively fill in these gaps by targeted disruption of these genes. Despite this, we have shown that this resource still provides an important means to probe ‘omics-based’ data. We therefore reasoned that direct access to this bank of mutants would be essential to enable a step change in the ease of reverse genetic studies and provide a major interface with other functional genomic resources and high-content data collections. We have therefore developed a REMI-seq website where all mutants in the GWDI-bank are catalogued and searchable by the gene ID (DDB_G0 number) or name of the disrupted gene. The website provides users with detailed instructions on how to order strains from the *Dictyostelium* stock centre [58] where each mutant has been deposited, as well as protocols for strain maintenance and validation of insertion points by diagnostic PCR.

We have also generated and characterised an extensive pool of sequenced ‘barcoded’ mutants in order to enable large-scale parallel phenotypic approaches. This can potentially be employed where any form of selection is placed on the population to enrich or deplete mutants that display a phenotype of interest. This is not restricted to growth as the library can also easily be transformed to express any reporter system that allows cells to selected (e.g., by FACS) for a response. The validated GWDI-pool has therefore been deposited in the *Dictyostelium* stock centre [58] and is available to order. In order to maximise the utility of this resource, we have provided detailed technical advice on performing genetic screens at the REMI-seq website, as well as step-by-step instructions and computational pipelines that take the user through the entire experimental procedure from ordering the pool, to performing the screens and then analysing the data. Sequencing of this library to an extremely high depth has allowed its complexity to be defined, thus representing a benchmark start point for any parallel phenotypic experiment.

We have used this library to perform selection experiments to identify and catalogue all mutants that exhibit changes in growth rate. These data represent an important resource because all parallel phenotypic experiments require the library to be grown on bacteria or liquid medium. Indeed, we find that a number of mutants that have previously been shown to exhibit defects in fluid uptake [59] can be found in the gridded library (e.g., *lst8*, *piaA*, *ripA*, *gacG*), but have either dropped out completely from the pool (*lst8*, *ripA*), are present at very low levels (*piaA*) or quickly drop out after further growth (*gacG*). We have also provided a sample dataset to allow users to familiarise themselves with the analysis process and thus facilitate the subsequent analysis of their own sequencing data. Different statistical methods for analysing the data, including by DESeq2 [60], are also presented. These data are freely available and thus represent an important resource for any lab undertaking parallel phenotypic analyses because it will always be important to distinguish mutants that are simply enriched or depleted due to growth defects, rather than due to the selection regime imposed.

These data highlight the power of parallel phenotypic approaches. For example, previous approaches to identify mutants that show growth defects have relied on laborious qualitative analysis of large numbers of mutant clones [52]. Interestingly, many of these are rescued by helper effects in mixed growth with wild-type cells [61]. The high throughput nature of REMI-seq, together with the fact that the pooled REMI-seq approach is refractive to identifying such mutants, likely explains why a different spectrum of mutants was identified. Consistent with this idea, the majority of mutants tested behave as predicted from pooled growth when assayed in clonal growth. Furthermore, it allowed the identification of novel biological pathways that mediate nutrient uptake, phagocytosis and macropinocytosis which may otherwise be missed if gene expression analyses are considered in isolation. Moreover, this provides further evidence that REMI-seq provides can be used to quantitatively assign phenotype to genotype in *D. discoideum*. Finally, these data provide an additional demonstration of how the integration of the gridded mutant set with the pools used for parallel phenotypic analysis provides an effective pipeline for functional genomic analyses in *D. discoideum*.

## Conclusion

The development of REMI-seq resources provides an important advance for high-throughput forward and reverse genetic identification of genes affecting traits associated with higher eukaryotes. Because their identification is often refractive to identification by other methods, REMI-seq provides a step change towards the goal of defining the genetic ‘components list’ underlying cell and developmental systems.

## Methods

### Growth and maintenance of strains

The axenic strain AX4 as well as all mutants generated in this strain was grown and maintained at 22°C either on SM plates (Formedium) spread evenly with *Klebsiella aerogenes* as a food source or in HL5 media containing glucose (Formedium) and 1x PVS on 10-cm tissue culture plates. 100x PVS comprises 3g penicillin G, 5g streptomycin sulphate, 10mg folic acid and 30mg vitamin B12 in 500ml H_2_0 and filter-sterilized and stored at 4°C in the dark.

### REMI mutagenesis

The GWDI insertion cassettes were amplified by PCR using T3 and T7 primers. pGWDI-G1, -G2 and -G3 have GATC-sticky ends generated by *BamH*I cleavage and can be used to insert at DpnII sires. pGWDI-C4, -G5, -C6, -C7 and -C8 have -CATG sticky ends generated by *Sph*I cleavage which can be used to insert at NlaII sites. The left and right arms of each vector can be distinguished by unique 6 bp indices (Additional file 3). Each vector also contains *I-Sce*I and *Mme*I sites that allow subsequent identification of insertion points. For transformation*,* log-phase cells were cooled for 15 min before collection by centrifugation for 2 min at 540g at 4°C. Cells were washed twice with cold KK2 + 50 mM sucrose before resuspending in the same buffer at 1.25x10^7^ cells/ml. 1.25x10^7^ cells were incubated with 10μg purified insert DNA and incubated on ice for 5 min. 1U enzyme was added to the mixture of cells, and insert DNA before 1x10^7^ cells were transformed using an ECM399 electroporation generator (900V, 150Ω, 36μF) in pre-cooled 4mm gap-width electroporation cuvettes. After transformation, 8μl of CaMg (100 mM CaCl_2_ and 100mM MgCl_2_) was added and cells were transferred to 60ml of filter-sterilised HL-5 medium including glucose, 10% horse serum and 1x PVS. 100μl of cells was added to each well of a 96-well dish and incubated at 22°C overnight, before blasticidin was added to a final concentration of 10μg/ml the following day. Cells were collected ~3–4 weeks later a grown up in HL5, spun down and resuspended in freezing medium (50% fetal bovine serum, 42.5% HL-5, 7.5% DMSO) and stored at −80°C. For growth, cells were thawed directly into filter-sterilised HL-5 media, and cells were allowed to recover for 24 h at 22°C.

### Preparation of DNA for Illumina sequencing

Details can be found on the REMI-seq.org website. Briefly, gDNA was extracted from cells bacterially grown cells. Nuclei were prepared by resuspending cells at around 1.25×10^7^ cells/ml in 40mM Tris, pH 7.8, 1.5% sucrose, 0.1mM EDTA, 6mM MgCl_2_, 40mM KCl, 0.4% NP-40 substitute and 5mM DTT, followed by centrifugation at 4000 g for 30min, 4°C. Nuclei were lysed by resuspension in 100mM EDTA and 5% sodium lauryl sarcosyl and incubation at 55°C for 20 min. After the addition of 2M, ammonium acetate debris was removed by centrifugation and DNA precipitated by the addition of absolute ethanol and mixed well. 0.5–1μg gDNA was digested with 10 U *Mme*I before digestion with 25U of *I-Sce*I (NEB), followed by precipitation and resuspension in 1x T4 DNA ligation buffer. Different combinations of D7 and D5 indexed adapters (2ng and 200ng, respectively) were ligated to the digested DNA (Additional file 3). Samples were then digested with *pshA*I to remove contaminating mitochondrial large subunit ribosomal RNA (*rnlA*) DNA. This was found to give a spurious band upon PCR amplification. DNA of interest was amplified by PCR using primers specific to the D7 and D5 adapters. DNA of interest was purified by two rounds of size selection before sequencing on a NextSeq® 500 Sequencer with a High Output Kit v2 (75 cycles).

### Analysis of Illumina sequence data

Each read was checked for the presence of the vector sequence [GC]AT[CG]CGTTGGA using an R Shiny app (https://github.com/NicoleGruenheit/REMI-seq-screen) after the 28bp adapter sequence was removed. From matching reads, the 6bp index sequence, which is located 13bp after the GATC or CATG, as well as the genomic tag including the *Dpn*II or *Nla*III site resulting in a 19/20bp tag sequence is extracted. Each tag and index combination are compared to a pre-computed lookup table of all potential genomic insertion points that also allows the number of tags to be counted. The orientation of each insert can also be determined from the combination of index sequence and genomic tag. The script generates the total number of reads per file, total number and percentage of reads that contain a tag, number of unique tags and number of tags in the inverted repeat (chromosome DDB0232429, 2263132 to 3015703 is repeated between bases 3016083 and 3768654), and number of non-unique tags. For information about grid layout and analysis, see https://github.com/NicoleGruenheit/grid_analyser.

### Cell motility assay

Cells were re-plated from their original GWDI-bank growth 96-well plates into a new grid at the lower density of 5 × 10^4^ cells per well. Once the cell had adhered to the plate surface, they were imaged on a CellInsight CX7 High-Content Screening (HCS) system and analysed using the cell motility package within the ThermoFischer HCS Studio Cell Analysis Software. For each strain, between 100 and 1000 individual cell images were captured at 2 min intervals for a minimum of 44 min. The distance travelled between each time point was calculated for each cell and then averaged (mean) for the whole population. Each time value was then summed to give the total path length for each strain. The measurements of two independent strains were averaged (mean) and compared to wild type. Significance differences were analysed using a *T* test.

### Selection for growth in axenic culture

Cells from the mutant library were seeded at a density of 2×10^5^ cells/ml on 10-cm tissue culture plates and allowed to grow around 36 h at 22°C (~4 generations) to reach confluency (3–4×10^6^ cells/ml) before being reseeded at 2×10^5^ cells/ml and grown for another 36 h. Cells were frozen down for every 72 h of continuous growth (~8 generations). Cells from a minimum of 5 plates were pooled and reseeded for each of the 2 biological replicates to mitigate bottlenecking of the mutant library (minimum 1×10^6^ cells reseeded = each mutant represented >40×). Each replicate was grown for a total of 78 generations. Genomic DNA was prepared from the mutant library following 24, 48 and 78 generations of axenic growth and processed for sequencing as described above.

### Selection for growth on *Klebsiella aerogenes*

The mutant library was initially thawed directly into filter-sterilised HL-5 media and allowed to recover for 24 h. Cells were then collected and washed twice in KK2 before being resuspended at 1×10^7^ cells/ml. 25μl of the suspension (2.5×10^5^) was then mixed with 400μl of an overnight culture of Ka and plated onto an SM plate. For each biological replicate of the screen, 4 plates were prepared in this way to prevent bottlenecking of the mutant library during serial transfer (1×10^6^ total cells = each mutant represented >40×). Following 48 h of growth (approximately 10 generations of growth), when the cells had eaten most of the bacteria but had not yet begun to aggregate and enter development, cells from all 4 plates were pooled, harvested and washed by repeated centrifugation at 500g in KK2 until all the bacteria had been removed. Cells were then resuspended at 1×10^7^ cells/ml, and 2.5×10^5^ cells were replated serially on fresh bacteria to begin another ‘round’ of selection. An aliquot of cells were also resuspended at 5×10^7^ cells/ml in freezing media and stored at −80°C.

### Analysis of mutant pools before and after selection

The mutant library was prepared for Illumina sequencing in triplicate as described above. Following sequencing, mutants were binned according to their mean normalised starting read counts (bin 100 ≤100 reads, bin 1000 = 100–1000 reads, bin 10,000 ≤1000 reads). Following sequencing of mutant pools after selection, logfold change values relative to starting read count were calculated for each insertion mutant (Additional file 8). To allow comparisons of mutants across time, we normalised these data to have a mean of 0 and a standard deviation of 1 for each of the 3 bins of mutants. Mutants with a mean *Z* score >1.5 (i.e., >1.5 standard deviations from the mean of that bin) were considered to have an advantage under that condition, and mutants with a mean *Z* score < -1 a disadvantage. Mutants with fewer than 100 starting read counts that dropped out were discounted from this analysis because the technical dropout rate for these mutants is very high (Additional file 6). Similarly, advantaged mutants required a minimum of 100 reads in both replicates at the end point of selection. Hierarchically clustered *Z* score data across time for each of the screens were visualised using the ggplot2 package in R [62].

### Growth mutant validation

A selection of axenic (7 mutants) and bacterial (12 mutants) mutants showing growth phenotypes was validated by growth competition assays with GFP-labelled AX4. To perform the growth competitions, cells of both AX4-GFP and competitor mutants were initially grown in parallel in tissue culture. Cells were then harvested, washed twice in KK2 and resuspended to 1×10^7^ cells/ml. To start each competition, mutant clones as well as the parental AX4 control were mixed 50:50 with AX4-GFP, and 2.5×10^5^ cells were then plated on either a single SM plate in association with 400μl *K. aerogenes* (bacterial growth competitions) or in tissue culture with 10mL HL5 (axenic competitions) in duplicate for 2 technical replicates per competition. Cells were allowed to grow together for approximately 20 (axenic) or 40 (bacterial) generations. The relative proportion of GFP-labelled to unlabelled cells was scored at the start as well as the end point of the competitions by flow cytometry (Attune NxT Flow cytometer). Competition data was normalised to 100% and 0% labelling controls as well as to wild-type labelled vs wild-type unlabelled controls. Data is expressed as a change in frequency from the start and the end of the competition (end frequency – start frequency, see Additional file 9) from at least 2 independent biological replicates.

### Fluid uptake assay

The fluid uptake assay was performed as described in [63], adapted to a 24-well dish format. Briefly, 2.5 × 10^5^ axenically growing cells were plated in triplicate on a 24-well dish. After settling onto the dish for 1 h, the HL5 media were aspirated and the cells incubated for 1 h with 0.5mg/ml TRITC-dextran (average Mw = 155kDa, Sigma Aldritch). The entire plate was then submerged and emptied twice in cold KK2 to quickly wash the cells, before cells from each well were collected in 1mL ice-cold KK2 + 5mM sodium azide to prevent continued exocytosis. Median fluorescence intensity was then measured by flow cytometry (Attune NxT Flow Cytometer). Values were adjusted for background by subtracting the unlabelled control and then normalised to control parental AX4 strain uptake (see Additional file 9), with at least 3 independent biological replicates performed per strain.

### Transcriptomes and GO analyses

To generate transcriptome data for axenically growing cells, we performed RNA-seq (Illumina). 2×10^7^ mid-logarithmic cells were collected and resuspended in 1mL TRIzol Reagent (Life Technologies) over consecutive days for a total of 3 replicates and stored at −80°C. After thawing, samples were mixed with 200μl chloroform, incubated for 2 min at room temperature and spun for 5min at 16,000rpm at 4°C. The colourless aqueous phase was separated and RNA precipitated in 500μl isopropanol, mixed and spun 10min at 16,000rpm at 4°C. The pellet was then washed in 1mL 70% ethanol and finally dried and resuspended in 40μl RNase-free ddH_2_0. RNA integrity was checked on an Agilent 2200 TapeStation. mRNA was then extracted by poly-A enrichment. RNA libraries were prepared using the Illumina TruSeq kit and sequenced using 100bp paired-end reads (150bp insert) on an Illumina Hiseq 4000. Reads were initially trimmed to remove remnants of Illumina TruSeq adapters and low-quality basepairs using the trimmomatic package [64]. After this, any reads of less than 20 bases were discarded, as were any in which the average quality of the sequence was less than a Phred score of 15. In all samples, more than 90% of the reads were retained. Trimmed sequences were then mapped to the AX4 *D. discoideum* reference genome using Bowtie2 [65]. A 750-kb duplication on chromosome 2 of AX4 was masked to ensure that reads mapping to the genes in this region were not filtered out subsequently because they do not map uniquely to the genome. Sensitive end-to-end mapping parameters were used requesting the 10 best matches. Finally, any reads that mapped multiple times to the genome were excluded, using the mapping quality flag in the resulting SAM Files. Mapped reads were sorted using SAMtools [66] and converted to BAMFiles before reads mapping to annotated genes were counted using the RPKM_count.py script from the RSeQC package [67].

To obtain bacterial growth transcriptome data, we downloaded the raw reads of RNA-seq data from the NCBI SRA database (accession numbers SRX271991 - SRX271998) for bacterially grown cells that used the same strain (AX4) under the same growth conditions as we used during the screen (clearing plates on SM agar in association with *K. aerogenes*) [68]. To ensure that differences in gene expression are not due to different methods to map and count the reads, we subjected the reads to the same pipeline used for the samples grown in HL5 (see above). We then filtered out low read count genes (<100 mean read counts in both axenic and *K. aerogenes* conditions) and calculated an axenic expression index by dividing the mean axenic read count by the sum of the mean read counts of both conditions (1 = expression exclusively on bacteria, 0 = expression exclusively axenic) to compare the expression level of genes in different conditions with the growth phenotype. To generate gene lists of differentially expressed genes, we performed differential gene expression analysis using the R package DESeq2 (version 1.14.1) [60]. Genes were considered to be differentially expressed if they showed >2 fold difference in expression and an adjusted *p* value <0.01 (Additional file 10).

To perform GO analyses on transcriptome and mutant lists, we used the GSEAbase R package [69] using a cutoff of *p*=0.05 for significantly over- or underrepresented GO terms. Gene lists for genes with phenotypes on *K. aerogenes* or HL5 were compared against a gene universe of genes from every mutant in the starting library. For differentially expressed gene lists, the gene universe comprised all annotated genes in the *D. discoideum* genome [26].

## Supplementary Information

**Additional file 1. **Lookup table for all possible insertion sites in the *D. discoideum* genome. Using the complete genome sequences of *Dictyostelium discoideum* the positions of all DpnII (GATC) and NlaIII (CATG) sites were identified as well as 20 bp up- and downstream of the recognition sites (genomic tags; Tab 1). Then, the number of occurrences of each tag in the genome as well as the location within or between genes was determined using this lookup table and the annotation File obtained from dictyBase. On top of this, the number of possible insertion sites was determined for each annotated gene (13,411; Tab 2) and all promoter regions (1,000 bp before the start codon). A short summary can be found in Tab 3.

**Additional file 2.** p-GWDI plasmids are derived from the pLPBLP plasmid. The sequence of the complete p-GWDI-G plasmid (4,488 bp) was annotated with the primers used for amplification of the insert as well as the left and right arms and the blasticidin resistance gene (bsr). Full sequence is available from REMI-seq.org.

**Additional file 3.** Indices present in different p-GWDI plasmids and Illumina adapter sequences. The ends of both arms contain indices specific for each arm and plasmid. p-GWDI G1, G2, G3 and G5 have GATC sticky ends and p-GWDI C4, C6, C7, C8 have CATG sticky ends.

**Additional file 4.** Table of individual mutants present in the REMI grid. Individual mutants were identified in 96-well plates using the REMI-seq technique (see methods). To identify the exact insertion points, the extracted tags were compared to the lookup table. The unique indices at the end of the inserts also allowed the determination of the vector version (p-GWDI G1 – G5; C6 – C8) as well as the orientation of the insert.

**Additional file 5.** Analyses of frequency of gene disruptions per site. Genes were grouped according to the number of possible insertions sites. The expected number of insertions for each gene was calculated based on a Poisson process and compared to the observed number of insertions in each gene.

**Additional file 6.** Mutants used in cell motility assays

**Additional file 7.** Table of individual mutants present in the pREMI pool. Individual mutants were identified after sequencing a pool of approximately 28,000 mutants using the REMI-seq technique. The pool was grown up and sequenced independently eight times.

**Additional file 8.** Read counts associated with each mutant are highly correlated between biological replicates from each round of selection after growth on bacteria (**A**) or in axenic medium (**B**).

**Additional file 9.** Identification of significantly enriched or depleted mutants from each round of selection after growth on bacteria (**A**) or in axenic medium (**B**) by Z score and read count cut-offs. The abundance (read count) at each round of each mutant was compared to the start pool. Mutants were first divided into bins based on their read count in the start pool in order to identify mutants that deviated in abundance (Z-score) significantly from other mutants with similar read counts. Mutants with a mean Z-score of >1.5 and with a minimum of 100 reads in each replicate at the end of the selection were considered to have increased in abundance (green points). Mutants with a mean Z-score of < -1.0 (red points) were considered to have decreased. In bin <100, the variation due to technical dropouts resulted in a high false discovery rate, and mutants that decreased were not considered. Replicas are highly correlated.

**Additional file 10.** Read count data before and after selections for growth in axenic culture or on bacteria. Normalised read count data is shown for each detectable insertion mutants for each replicate of each condition, as well as log fold change values from the starting read count before the selections.

**Additional file 11.** List of mutations and associated genes that affect growth on bacteria or in axenic medium when disrupted.

**Additional file 12.** Growth competitions and fluid uptake assays data

**Additional file 13.** Differential gene expression analysis on axenic vs bacterial growth

**Additional file 14.** GO term analysis of genes associated with growth in axenic culture or on bacteria

## Data Availability

Raw reads of all samples were uploaded to the NCBI SRA database (PRJNA524784 [70] and PRJNA524539 [71]. The R Shiny apps to analyse pools of REMI-seq mutants or grids can be found here https://github.com/NicoleGruenheit/REMI-seq-screen [72] and here: https://github.com/NicoleGruenheit/grid_analyser [73]. Detailed protocols, computational pipelines and further information can also be found at www.remi-seq.org.
