# Supplementary material for: Mutant resources for functional genomics in Dictyostelium discoideum using REMI-seq technology
Source: BMC Biol. 2021 Aug 24;19:172. doi: 10.1186/s12915-021-01108-y (PMC8386026; doi:10.1186/s12915-021-01108-y)
Supplement: Supplementary file 2 — Additional file 2. p-GWDI plasmids are derived from the pLPBLP plasmid. The sequence of the complete p-GWDI-G plasmid (4,488 bp) was annotated with the primers used for amplification of the insert as well as the left and right arms and the blasticidin resistance gene (bsr). Full sequence is available from REMI-seq.org. [file 12915_2021_1108_MOESM2_ESM.pdf]

Created with SnapGene®

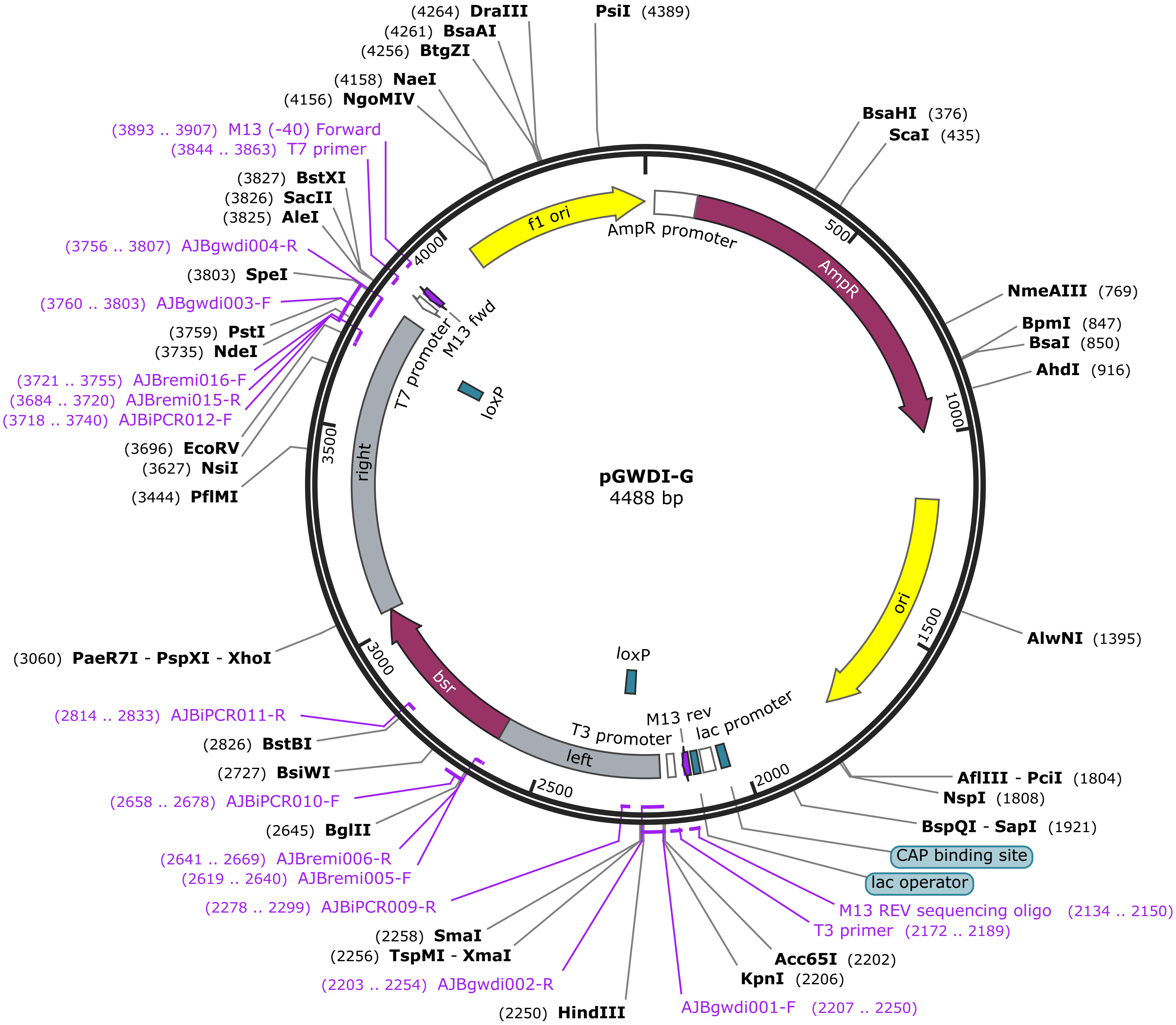

gtggcacttttcggggaaatgtgcgcggaacccctatttgtttatttttctaaatacattcaaatatgtatccgctCATG
agacaataaccctgataaatgcttcaataatattgaaaaaggaagagtatgagtattcaacatttccgtgtcgcccttat
tcccttttttgcggcattttgccttcctgtttttgctcacccagaaacgctggtgaaagtaaaagatgctgaaGATCagt
tgggtgcacgagtgggttacatcgaactgGATCtcaacagcggtaaGATCcttgagagttttcgccccgaagaacgtttt
ccaatgatgagcacttttaaagttctgctatgtggcgcggtattatcccgtattgacgccgggcaagagcaactcggtcg
ccgcatacactattctcagaatgacttggttgagtactcaccagtcacagaaaagcatcttacggatggCATGacagtaa
gagaattatgcagtgctgccataacCATGagtgataacactgcggccaacttacttctgacaacGATCggaggaccgaag
gagctaaccgcttttttgcacaaCATGgggGATCatgtaactcgccttGATCgttgggaaccggagctgaatgaagccat
accaaacgacgagcgtgacaccacgatgcctgtagcaatggcaacaacgttgcgcaaactattaactggcgaactactta
ctctagcttcccggcaacaattaatagactggatggaggcggataaagttgcaggaccacttctgcgctcggcccttccg
gctggctggtttattgctgataaatctggagccggtgagcgtgggtctcgcggtatcattgcagcactggggccagatgg
taagccctcccgtatcgtagttatctacacgacggggagtcaggcaactatggatgaacgaaatagacaGATCgctgaga
taggtgcctcactgattaagcattggtaactgtcagaccaagtttactcatatatactttagattgatttaaaacttcat
ttttaatttaaaagGATCtaggtgaaGATCctttttgataatctCATGaccaaaatcccttaacgtgagttttcgttcca
ctgagcgtcagaccccgtagaaaaGATCaaagGATCttcttgaGATCctttttttctgcgcgtaatctgctgcttgcaaa
caaaaaaaccaccgctaccagcggtggtttgtttgccgGATCaagagctaccaactctttttccgaaggtaactggcttc
agcagagcgcagataccaaatactgtccttctagtgtagccgtagttaggccaccacttcaagaactctgtagcaccgcc
tacatacctcgctctgctaatcctgttaccagtggctgctgccagtggcgataagtcgtgtcttaccgggttggactcaa
gacgatagttaccggataaggcgcagcggtcgggctgaacggggggttcgtgcacacagcccagcttggagcgaacgacc
tacaccgaactgagatacctacagcgtgagctatgagaaagcgccacgcttcccgaagggagaaaggcggacaggtatcc
ggtaagcggcagggtcggaacaggagagcgcacgagggagcttccagggggaaacgcctggtatctttatagtcctgtcg
ggtttcgccacctctgacttgagcgtcgatttttgtgatgctcgtcaggggggcggagcctatggaaaaacgccagcaac
gcggcctttttacggttcctggccttttgctggccttttgctcaCATGttctttcctgcgttatcccctgattctgtgga
taaccgtattaccgcctttgagtgagctgataccgctcgccgcagccgaacgaccgagcgcagcgagtcagtgagcgagg
aagcggaagagcgcccaatacgcaaaccgcctctccccgcgcgttggccgattcattaatgcagctggcacgacaggttt
cccgactggaaagcgggcagtgagcgcaacgcaattaatgtgagttagctcactcattaggcaccccaggctttacactt
tatgcttccggctcgtatgttgtgtggaattgtGAGCGGATAACAATTTCACACAGGaaacagctatgacCATGattacg
ccaagcgcgcaattaaccctcactaaagggaacaaaagctgggtaccgGATCcGTTGGActgctgCGTGATtagggataa
CAGGGTAATaagcttcccgggttaattaattaaccacggataacttcgtatagcatacattatacgaagttataGATCct
ctagataaaaaaaatttttatttatttttatttattttgaattaaatagattacaaattaattaatcccatcaaatcttt
aaaaaaaaaaaatggtttaaaaaaacttgggttggttaattattatttgaaaattttaaaacccaaattaaaaaaaaaaaa
tgggattcaaaaattttttttttttttttttttttttttttttttttttttttttttttcagattgcataaaaagattttt
tttttttttttttcttatttcttaaaacaaataaattaaattaaataaaaaaaataaaaATGGATCAATTTAACATTTCTC
AACAAGATCTAGAATTAGTAGAAGTAGCGACAGAGAAGATTACAATGCTTTATGAGGATAATAAAC
ATCATGTGGGAGCG
GCAATTCGTACGAAAACAGGAGAAATCATTTCGGCAGTACATATTGAAGCGTATATAGGACGAGTA
ACTGTTTGTGCAGA
AGCCATTGCGATTGGTAGTGCAGTTTCGAATGGACAAAAGGATTTTGACACGATTGTAGCTGTTAGA
CACCCTTATTCTG
ACGAAGTAGATAGAAGTATTCGAGTGGTAAGTCCTTGTGGTATGTGTAGGGAGTTGATTTCAGACT
ATGCACCAGATTGT
TTTGTGTTAATAGAAATGAATGGCAAGTTAGTCAAAACTACGATTGAAGAACTCATTCCACTCAAAT
ATACCCGAAATTA
Aaagttttaccataccaagctcgagctcgctagagtcgtccatcaattgttcacagaaaatgtttctaaattatttaata
aataataaaaaaacaaattgttgtaataatctaatattttcttttttttttaatttttttttttttaaatcttaataatta
ttaagttattttaattttttttttttttttttttttttttttttcccaccaaaaaaaccaaatatttttaaaaattttt
ttttttacaaaaactttggagggggaaaaaaaaaaattggcttaaaaggaaaaccccccaaaaagaagaaaaaccaaaa

aaaaaaggcggacccaaaagccccccatcattggtccaaaaaaagtgttcctaattaatttaaaaaaaaaaaaaaaacca
attggtggaaaaactcaaaaattttctttttttttaaattttttttttttaaaatctaaaattaataaggtaatttaattt
tttttttttttttttttttctttttttttttctatcaaaaaaaatcaaatatatttaaaaaatttattatttacagatacat
tttgaatggtgaagataaatatatgcattagatgtaaaacagccaaagagtatgaaaatcaaaaagataaagctgacccg
aaagctcgGATCtgatatcataacttcgtatagcatacattatacgaagttatcatatgccgCATGgttaattcctgcag
attaccctgttatCCCTACGATGTcagcagTCCAACgGATCcactagtctagagcgccgccaccgcggtggagctccaat
tcgccctatagtgagtcgtattacgcgcgctcactggccgtcgttttacaacgtcgtgactgggaaaaccctggcgttac
ccaacttaatcgccttgcagcacatccccctttcgccagctggcgtaatagcgaagaggcccgcaccGATCgcccttccc
aacagttgcgcagcctgaatggcgaatgggacgcgccctgtagcggcgcattaagcgcggcgggtgtggtggttacgcgc
agcgtgaccgctacacttgccagcgccctagcgcccgctcctttcgctttcttcccttcctttctcgccacgttcgccgg
ctttccccgtcaagctctaaatcgggggctccctttagggttccgatttagtgctttacggcacctcgaccccaaaaaac
ttgattagggtgatggttcacgtagtgggccatcgccctgatagacggtttttcgccctttgacgttggagtccacgttc
tttaatagtggactcttgttccaaactggaacaacactcaaccctatctcggtctattcttttgatttataagggatttt
gccgatttcggcctattggttaaaaaatgagctgatttaacaaaaatttaacgcgaattttaacaaaatattaacgctta
caatttag
