# Supplementary material for: Mutant resources for functional genomics in Dictyostelium discoideum using REMI-seq technology
Source: BMC Biol. 2021 Aug 24;19:172. doi: 10.1186/s12915-021-01108-y (PMC8386026; doi:10.1186/s12915-021-01108-y)
Supplement: Supplementary file 6 — Additional file 6. Mutants used in cell motility assays [file 12915_2021_1108_MOESM6_ESM.docx]

| Supplementary File 6   \| **Gene name** \| **Putative Function** \| \| --- \| --- \| \|  \|  \| \| kxcB \| serine/threonine protein kinase with RhoGEF, DH and PH domains \| \| docA \| DOCK180 protein homologue \| \| zizA \| zizimin- related DOCK family protein member \| \| vilC \| villin-like protein C \| \| elmoE \| ElmoE forms a complex with DocC and ZizA \| \| rapgap1 \| **Rap**GTPase, **G**uanosine triphosphatase-**A**ctivating **P**rotein 1 \| \| Roco10 \| LRRK family protein kinase \| \|  \|  \| \| DDB_G0277675 \| RasGTPase-activating protein \| \| DDB_G0283827 \| WASP-related protein C \| \| DDB_G0289829 \| Unknown \| \| DDB_G0277997 \| Neuroblastoma-amplified sequence \| \| DDB_G0277165 \| CAMKL homologue, BRSK protein kinase subfamily \| |
| --- | --- | --- | --- | --- | --- | --- | --- | --- | --- | --- | --- | --- | --- | --- | --- | --- | --- | --- | --- | --- | --- | --- | --- | --- | --- | --- | --- | --- | --- | --- |
