# Supplementary figures and images for: Mutant resources for functional genomics in Dictyostelium discoideum using REMI-seq technology
Source: BMC Biol. 2021 Aug 24;19:172. doi: 10.1186/s12915-021-01108-y (PMC8386026; doi:10.1186/s12915-021-01108-y)

**A**

**Ka 100 generations**

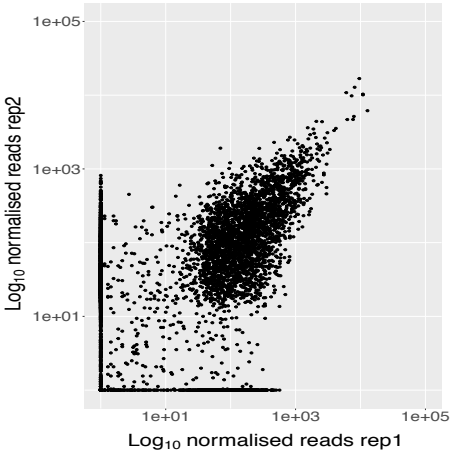

**B**

**Axenic 24 generations**

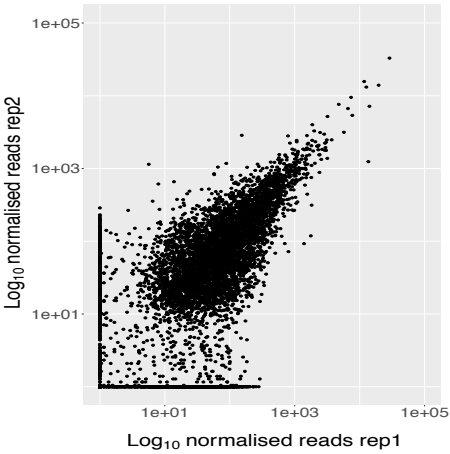

**Ka 200 generations**

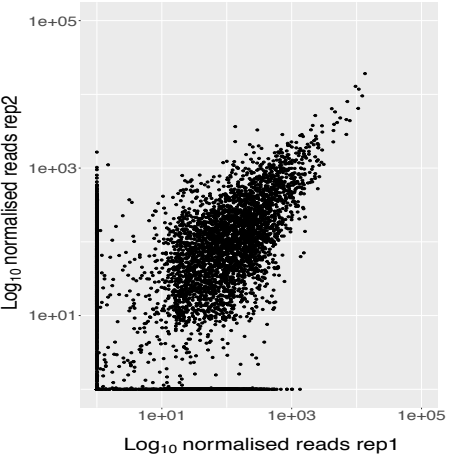

**Axenic 48 generations**

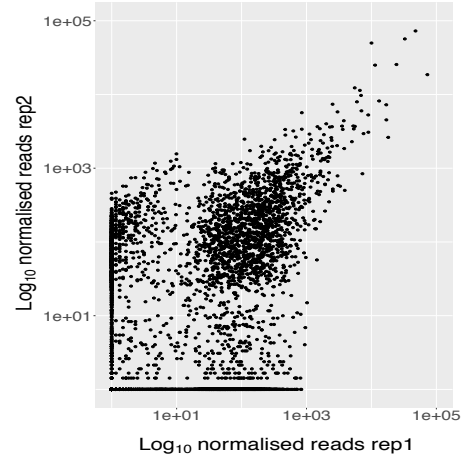

**Axenic 72 generations**

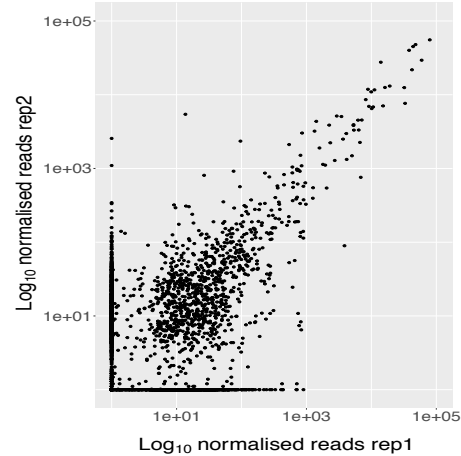

Supplement: Supplementary file 8 — Additional file 8. Read counts associated with each mutant are highly correlated between biological replicates from each round of selection after growth on bacteria (A) or in axenic medium (B). [file 12915_2021_1108_MOESM8_ESM.pdf]
