# Supplementary material for: Mutant resources for functional genomics in Dictyostelium discoideum using REMI-seq technology
Source: BMC Biol. 2021 Aug 24;19:172. doi: 10.1186/s12915-021-01108-y (PMC8386026; doi:10.1186/s12915-021-01108-y)

Supplementary file 9

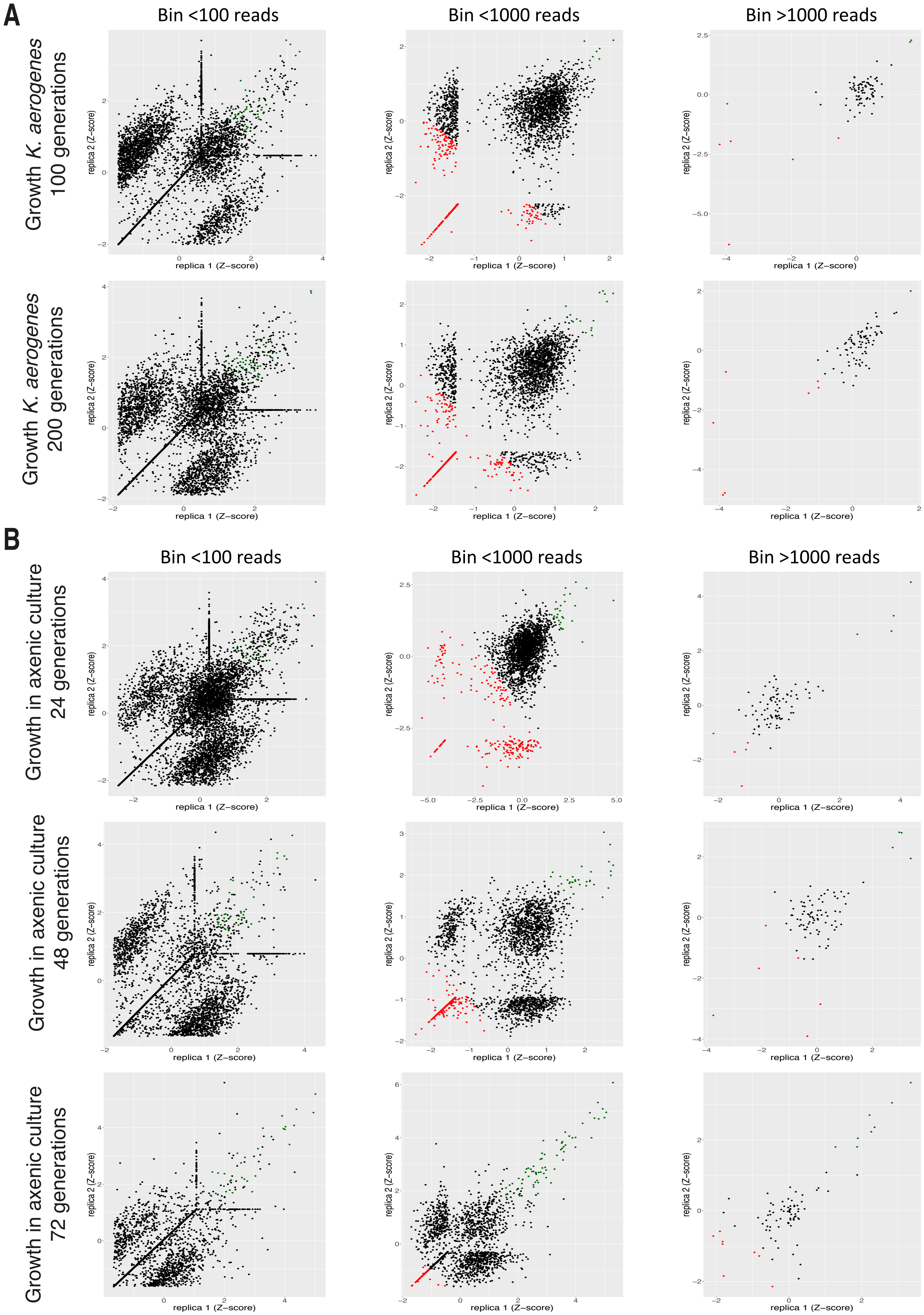

Supplement: Supplementary file 9 — Additional file 9. Identification of significantly enriched or depleted mutants from each round of selection after growth on bacteria (A) or in axenic medium (B) by Z score and read count cut-offs. The abundance (read count) at each round of each mutant was compared to the start pool. Mutants were first divided into bins based on their read count in the start pool in order to identify mutants that deviated in abundance (Z-score) significantly from other mutants with similar read counts. Mutants with a mean Z-score of >1.5 and with a minimum of 100 reads in each replicate at the end of the selection were considered to have increased in abundance (green points). Mutants with a mean Z-score of < -1.0 (red points) were considered to have decreased. In bin <100, the variation due to technical dropouts resulted in a high false discovery rate, and mutants that decreased were not considered. Replicas are highly correlated. [file 12915_2021_1108_MOESM9_ESM.pdf]
